# Endosialin in Cancer: Expression Patterns, Mechanistic Insights, and Therapeutic Approaches

**DOI:** 10.7150/thno.89495

**Published:** 2024-01-01

**Authors:** Shiqi Lu, Lunbiao Gan, Tong Lu, Keying Zhang, Jiayu Zhang, Xinjie Wu, Donghui Han, Chao Xu, Shaojie Liu, Fa Yang, Weijun Qin, Weihong Wen

**Affiliations:** 1 Xi'an Key Laboratory of Stem Cell and Regenerative Medicine, Institute of Medical Research, Northwestern Polytechnical University, Xi'an, Shaanxi 710072, China.; 2 Department of Urology, Xijing Hospital, Fourth Military Medical University, Xi'an, Shaanxi 710032, China.

**Keywords:** endosialin, TEM1, CD248, tumor progression, angiogenesis, tumor microenvironment, targeted therapy

## Abstract

Endosialin, also known as tumor endothelial marker 1 (TEM1) or CD248, is a single transmembrane glycoprotein with a C-type lectin-like domain. Endosialin is mainly expressed in the stroma, especially in cancer-associated fibroblasts and pericytes, in most solid tumors. Endosialin is also expressed in tumor cells of most sarcomas. Endosialin can promote tumor progression through different mechanisms, such as promoting tumor cell proliferation, adhesion and migration, stimulating tumor angiogenesis, and inducing an immunosuppressive tumor microenvironment. Thus, it is considered an ideal target for cancer treatment. Several endosialin-targeted antibodies and therapeutic strategies have been developed and have shown preliminary antitumor effects. Here, we reviewed the endosialin expression pattern in different cancer types, discussed the mechanisms by which endosialin promotes tumor progression, and summarized current therapeutic strategies targeting endosialin.

## 1. Introduction

Although the incidence of some cancer types has decreased in recent years, cancer is still a serious threat to human health. Most tumors are difficult to discover in the early stage, while in the late stage, tumors may progress rapidly, and metastasis may occur 1, 2. Current strategies for cancer treatment cannot meet clinical requirements, and the antitumor effect of some treatment strategies is not satisfactory. Therefore, it is important to elucidate the mechanisms of tumor development and progression and identify novel tumor-specific markers for early diagnosis and treatment.

The tumor microenvironment (TME) is composed of various cellular and acellular components, including immune cells, stroma cells, blood vessels, extracellular matrix (ECM) proteins and many secretory molecules 3. Among them, cancer-associated fibroblasts (CAFs) have been found to promote tumor progression through direct mechanisms (by cell-to-cell communication) or indirect mechanisms (through secretion of soluble factors or extracellular vesicles) 4-8. Thus, CAFs are considered suitable therapeutic targets for cancer treatment. However, until now, no ideal markers have been identified for CAF-specific targeting. Furthermore, CAFs can be divided into different subtypes, and some subtypes may even have antitumor functions 9, 10. Therefore, it is important to identify biomarkers that are specifically expressed in CAFs and elucidate whether they are important for tumor progression, which will contribute to the development of novel promising therapeutic strategies.

Endosialin, also known as tumor endothelial marker 1 (TEM1) or CD248, is a type I transmembrane glycoprotein that belongs to the C-type lectin domain (CTLD) group 14 family of transmembrane glycoproteins 11. Endosialin was first identified as a tumor stromal antigen by using FB5 antibody, which was generated through immunization with human fibroblasts fused with myeloma cells. It was named endosialin and was found to be selectively expressed in tumor endothelial cells but not in normal blood vessels or other adult tissues 12. The human endosialin gene was cloned in 2001 and was found to have the same sequence as TEM1. Endosialin is composed of a signal leader peptide, five globular extracellular domains (including a C-type lectin domain, one domain with similarity to the Sushi/ccp/scr pattern, and three EGF repeats), followed by a mucin-like region, a transmembrane segment, and a short cytoplasmic tail 13. In 2001, when St Croix et al. tried to identify the differentially expressed genes in tumor endothelial cells, they compared the gene expression patterns in endothelial cells derived from blood vessels of normal and malignant colorectal tissues and found that endosialin was one of the genes that was specifically expressed in tumor endothelial cells but barely expressed in normal tissues 14.

Until now, several molecules have been identified to bind with endosialin; these include MMRN2, Mac-2BP/90K, galectin-3, CD68, and some ECM proteins, such as fibronectin (FN), collagen types I and IV (Col I and Col IV) 11, 15. Studies have revealed that endosialin can promote tumor progression through multiple mechanisms, such as promoting tumor cell proliferation, adhesion and migration, stimulating tumor angiogenesis and inducing an immunosuppressive TME. Because of its tumor-promoting function, endosialin is considered an ideal target for cancer treatment.

In this review, we summarized the expression pattern of endosialin in different cancer types, elucidated its tumor-promoting mechanism, and discussed the research progress regarding endosialin-targeted therapeutic strategies.

## 2. Endosialin expression in different cancer types

### 2.1 Endosialin is highly expressed in CAFs and pericytes in epithelial cell-derived tumors and correlated with patient prognosis

Although endosialin was initially found to be selectively expressed in endothelial cells of various tumors, subsequent studies clarified that endosialin was mainly expressed in the tumor stroma, especially CAFs and pericytes. For example, Rouleau et al. examined endosialin expression in 250 clinical specimens of human cancer, including 20 cancer subtypes, and found that endosialin was mainly expressed in stromal cells and perivascular cells in carcinomas 16. By using four newly generated antibodies, MacFadyen et al. demonstrated that endosialin was predominantly expressed in fibroblasts and a subset of tumor vessel-associated pericytes but not in the tumor endothelium 17. Christian et al. also found that endosialin was expressed in CAFs and tumor vessel-associated mural cells but not endothelial cells 18.

Other studies have also examined endosialin expression in different cancer types. In melanoma, Kiyohara et al. found that 70% (46/66) of stage III or stage IV melanoma specimens and 86% (117/136) of stage IV specimens had endosialin expression, mainly in pericytes and stromal fibroblasts, while no expression was detected in 29 normal tissue controls 19. Huber et al. reported that in cutaneous melanoma metastases and squamous cell carcinomas, endosialin was predominantly expressed either in tumor blood vessels or both tumor blood vessels and stromal fibroblasts 20.

In brain tumor, several studies reported inconsistent results. Carson-Walter et al. found that endosialin expression was upregulated in primary and metastatic human brain tumors, and it was primarily localized to the tumor vasculature and a subset of tumor stromal cells 21. Brady et al. found that endosialin was mainly expressed in highly invasive glioblastoma multiforme, anaplastic astrocytomas and metastatic carcinomas; and endosialin was localized to the endothelium of small and large vessels, Thy-1-positive fibroblast-like cells and α-smooth muscle actin (α-SMA)-positive cells 22. However, Simonavicius et al. demonstrated that endosialin expression was upregulated in high-grade gliomas and was mainly expressed in tumor-associated pericytes but not endothelial cells 23. While in another study, Rouleau, et al. examined endosialin expression in neuroblastoma, small cell lung cancer and melanoma, and they observed vascular endosialin staining in all three kinds of tumors. Interestingly, they found that tumor cells also expressed endosialin, and the expression was highest in neuroblastoma, weak in melanoma and rare in small cell lung cancer 24.

In gastric cancer, Fujii et al. examined endosialin expression in a tissue microarray that contained 945 tumor tissues and found that endosialin was specifically expressed in CAFs, and its expression was significantly correlated with recurrence-free survival, overall survival, cancer-related overall survival, scirrhous subtype, tumor depth, nodal status, distant metastasis, serosal invasion, lymphatic or venous vessel infiltration and pTMN stage 25. In colorectal cancer (CRC), O'Shannessy et al. found that stromal expression of endosialin had prognostic value, and signature combining endosialin expression score with other compartment-specific expression scores (endosialin stroma, endosialin tumor vessel, HIF2α stromal vessel, Col IV tumor, and FN stroma) had even better prognostic value, specifically in stage II CRC patients 26.

In breast cancer, Davies et al. found that patients with recurrent disease or those who died of breast cancer had a significantly elevated expression of endosialin, and the elevated endosialin level was associated with nodal involvement and disease progression 27. In ovarian cancer, when Kuk et al. tried to identify potential biomarkers from soluble ascites, they found that endosialin was among the 52 new proteins that deserve further clinical validation 28. In RCC, Xu et al. found that endosialin-positive CAFs were correlated with poor prognosis and an immunosuppressive TME 29.

In addition to its specific expression on CAFs and pericytes, endosialin was also found to be expressed in the serum of cancer patients. For example, Pietrzyk et al. found that serum level of endosialin was significantly higher in CRC patients than in healthy controls, and high endosialin level was associated with CRC progression and a poor prognosis 30. However, in another study, O'Shannessy et al. examined the serum level of endosialin using novel antibodies they generated, yet they found that the serum level of endosialin was not different between CRC patients and healthy individuals 31. Thus, whether it could be used as a serum biomarker for cancer needs to be further evaluated.

### 2.2 Endosialin is also highly expressed in tumor cells of mesenchymal cell-derived sarcomas

In addition to its expression in the tumor stroma of epithelial cell-derived cancer types, endosialin was also found to be highly expressed in the tumor cells of mesenchymal cell-derived sarcomas. In Rouleau's study, they found that in sarcoma tissue, not only tumor stromal cells and perivascular cells, but also tumor cells had endosialin expression 16. Guo et al. analyzed endosialin expression in 19 human sarcoma subtypes (203 specimens) and found that endosialin was expressed in 96% of human sarcomas, among which 81% had endosialin expression in both tumor cells and tumor vasculature 32. O'Shannessy et al. examined endosialin expression in a cohort of 94 sarcoma patients and found that endosialin was highly expressed and that its expression was positively correlated with the expression level of platelet-derived growth factor receptor-β (PDGFR-β) 33.

Thway et al. examined endosialin expression in 514 human soft tissue sarcomas and found that endosialin was expressed in 89% of undifferentiated pleomorphic sarcomas (104/117), 77% of fibrosarcomas (20/26), 62% of synovial sarcomas (37/60), 51% of leiomyosarcoma (94/185) and 31% of rhabdomyosarcoma (39/126). These findings indicate that endosialin could be used to distinguish undifferentiated and poorly differentiated sarcomas 34. De Gooyer et al. found that strong endosialin expression was common (88.2%) in myxofibrosarcoma and that endosialin could be used as a suitable target for tumor-targeted imaging 35.

Some studies have also found that endosialin expression is closely correlated with tumor malignancy. For example, Kondo et al. found that in 10 cases of nonmetastatic osteosarcoma (OS), only one had endosialin expression, while 7 of the 8 metastatic OSs had endosialin expression, indicating that high endosialin expression was correlated with metastasis 36. Rouleau et al. also conducted a retrospective analysis of clinical specimens and found that all high-grade and metastatic sarcomas had higher endosialin expression than low-grade sarcomas 37.

The side population (SP) is considered to have stem cell-like properties, and Rouleau et al. found that endosialin was expressed in the SP of sarcoma cell lines 38. Sun et al. also found that primary human OS samples contained approximately 3.9% SP cells, which are responsible for therapy failure and tumor recurrence. These endosialin-positive SP cells were able to regenerate the tumor population and had high invasive potential, indicating that endosialin-positive SP cells might be a potential target to prevent OS recurrence after chemotherapy 39.

Therefore, in epithelial cell-derived cancers, endosialin was mainly expressed in stromal cells, especially CAFs and pericytes, while in mesenchymal cell-derived sarcomas, endosialin was also expressed in tumor cells. Because of its specific high expression in different cancer types, endosialin is considered to be an effective therapeutic target for cancer treatment 40.

## 3. Mechanisms of how endosialin promotes tumor progression

### 3.1 Promoting tumor cell proliferation, adhesion and migration

The tumor-promoting function of endosialin was first demonstrated in endosialin knockout (KO) mice. It was shown that there was no difference in the growth of subcutaneously inoculated tumors in endosialin KO mice; however, tumor growth, invasiveness and metastasis were significantly inhibited in endosialin KO mice when tumor cells were transplanted in abdominal sites, indicating that endosialin has an anatomical site-specific tumor-promoting function or that the local microenvironment might be involved in endosialin-mediated tumor progression 41. In endosialin transgenic mice, which express endosialin lacking its cytoplasmic domain, the growth of T241 fibrosarcoma and Lewis lung carcinoma was significantly reduced compared with wild-type (WT) mice. In addition, compared with WT fibroblasts, conditioned medium from fibroblasts from the same transgenic mice showed impaired supportive function for tumor cell survival, indicating that the cytoplasmic domain of endosialin is critical for its tumor-promoting function, possibly through other intracellular interacting proteins and/or downstream signaling pathways 42.

Since endosialin is highly expressed in the tumor stroma, it is speculated that endosialin may promote cell adhesion and migration of epithelial cell-derived tumors through cell-cell or cell-ECM interactions. One study showed that stromal fibroblast-expressed endosialin could bind with Mac-2BP/90K, which is highly expressed in tumor cells; thus, endosialin might promote tumor cell adhesion and migration through interaction with Mac-2BP/90K 43. Another study showed that endosialin-expressing pericytes could promote tumor cell intravasation in a cell contact-dependent manner, thus facilitating distant metastasis. They also showed that in breast cancer patients, upregulated endosialin levels were significantly correlated with increased metastasis and poor prognosis 44. For ECM proteins, endosialin was found to bind with FN, Col I and Col IV, and the interaction could be blocked by the anti-endosialin antibody MORAb-004; overexpression of endosialin in CHO cells enhanced cell adhesion to FN and promoted cell migration in Matrigel, indicating that endosialin may promote tumor progression and invasion 45.

In colon cancer, Park et al. found that endosialin could regulate cell migration and drug resistance; overexpression of endosialin could promote cell migration, while downregulation of endosialin resulted in increased cell apoptosis in chemotherapy-resistant cells 46. In breast cancer, Huang et al. constructed a bone metastasis-specific regulatory network based on prognostic stemness-related signatures (PSRSs), their upstream transcription factors (TFs) and downstream pathways and found that MAF may positively regulate endosialin expression and that endosialin may influence breast cancer bone metastasis via the apical junction pathway 47.

Endosialin expressed in mesenchymal cell-derived sarcoma cells has also been shown to promote cell proliferation and migration. It was found that overexpression of endosialin in endosialin-negative osteosarcoma MG63 cells could significantly promote cell proliferation and migration 48. Lu et al. found that knockdown of endosialin significantly inhibited OS cell migration, invasion and lung metastasis but had no obvious effect on cell proliferation *in vitro* or tumor growth *in vivo*. Mechanistic study showed that CD248 could promote the interaction between ITGB1 and ECM proteins and activate the FAK-paxillin pathway to promote the formation of focal adhesion and metastasis of OS cells 49. Since the humanized endosialin antibody MORAb-004 could effectively inhibit the migration of sarcoma cells but had no obvious effect on cell proliferation, endosialin was considered to be involved in the metastatic process of OS 36.

**Figure 1:**
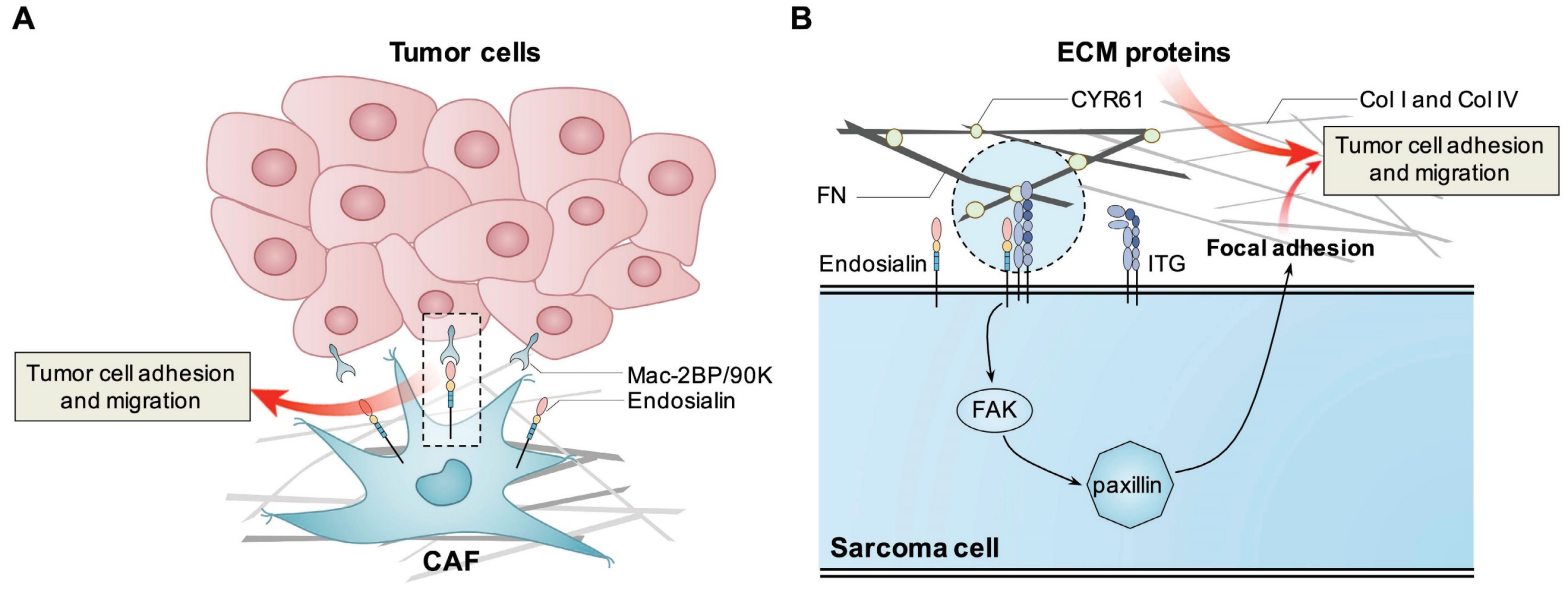
** Known mechanisms how endosialin promotes tumor cell proliferation, adhesion and migration.** Endosialin expressed in CAFs binds with Mac-2BP/90K expressed in tumor cells to promote tumor cell adhesion and migration. Tumor cell expressed endosialin could bind with ECM proteins like FN, Col I and Col IV to enhance cell adhesion and migration, or promote the interaction between ITGB1 and ECM proteins, activate the FAK-paxillin pathway, and promote the formation of focal adhesion and metastasis.

Thus, endosialin can promote tumor progression through different mechanisms. In epithelial cell-derived tumors, endosialin may promote tumor cell proliferation, adhesion and migration through cell-cell or cell-ECM interactions, while in mesenchymal cell-derived sarcomas, endosialin may promote tumor cell migration and invasion through intracellular pathways such as the FAK-paxillin pathway (summarized in Figure 1). However, the detailed molecular mechanisms have not been fully elucidated, and more studies are still needed.

### 3.2 Stimulating tumor angiogenesis

In addition to the regulation of tumor cells, endosialin was also found to be involved in tumor angiogenesis 50. For example, Bagley et al. found that endosialin was expressed in tumor vasculature pericytes and that an endosialin antibody could inhibit pericyte tube formation and migration *in vitro*, indicating that endosialin was involved in active angiogenesis during tumor development 51. In addition, they also found that endosialin was also highly expressed in endothelial precursor cells (EPCs) than in mature endothelial cells, and anti-endosialin antibodies inhibited EPC migration and tube formation *in vitro* and decreased the number of circulating murine EPCs in tumor-bearing mice. These findings indicated that endosialin is involved in the earlier stages of tumor angiogenesis 52.

The proangiogenic function of endosialin was also demonstrated in KO and knock-in mice. Nanda et al. found that in endosialin KO mice, wound healing was normal, indicating that endosialin is not required for neovascularization during wound repair.

However, tumor vessels failed to efficiently mature, leading to decreased numbers of medium and large vessels and a compensatory increase in small vessels, indicating that endosialin is needed for efficient maturation of vessels within tumors 41. By using an orthotopic lung cancer model, Hong et al. also found that in endosialin KO mice, tumor volume, the density of vessels and pericytes, and the functionality of tumor vessels were all decreased. Mechanistically, they found that endosialin could activate Wnt/β-catenin signaling and upregulate two angiogenic factors, OPN and SERPINE1, in pericytes, resulting in enhanced angiogenesis and lung cancer growth 53. In human endosialin knock-in mice, Rybinski et al. found that MORAb-004 could induce the internalization of endosialin into pericytes and impair tumor microvasculature maturation, thus inhibiting tumor growth and metastasis 54.

In renal cell carcinoma (RCC), endosialin was found to be specifically expressed in blood vessels, and its expression level was found to be correlated with microvascular density 55. In urothelial carcinoma of the bladder (UCB), endosialin was also found to be specifically expressed in blood vessels, and its expression was closely associated with a poor prognosis in UCB patients 56. By using a mouse model composed of tumor cells and endosialin-expressing endothelial cells, Li et al. demonstrated that the endosialin antibody MORAb-004 could inhibit tumor angiogenesis, indicating that endosialin was involved in tumor vasculature 57.

In pancreatic cancer, endosialin was demonstrated to bind with MMRN2, which is a unique endothelial-specific ECM protein that has been implicated in angiogenesis and tumor progression. Endosialin and CLEC14A, which is another C-type lectin domain-containing group 14 family member, could simultaneously bind with MMRN2 at the interface between the endothelium and pericytes in human pancreatic cancer and were speculated to promote tumor angiogenesis 58. In addition, Tomkowicz et al. found that endosialin played a role in PDGF-induced proliferation of vascular pericytes. When endosialin was knocked down, PDGF-BB-induced proliferation, ERK1/2 phosphorylation, and c-Fos expression were significantly suppressed. Thus, targeting endosialin and the PDGF/ERK1/2/c-Fos pathway may provide novel strategies to inhibit tumor angiogenesis 59. Interestingly, Hong et al. reported that during wound healing, endosialin expression was highly upregulated, and endosialin could bind with PDGFR to enhance the mitogenic and chemoattractive effects of PDGF-BB and collagen deposition in myofibroblasts, thus promoting wound healing 60. In another study, Brett et al. treated mice with dermal wounds with the sorted endosialin positive stromal vascular fraction (SVF) from human lipoaspirate, and found that wounds healed significantly faster than the endosialin negative or unsorted SVF groups. These data also indicates that endosialin has pro-angiogenic function 61.

**Figure 2:**
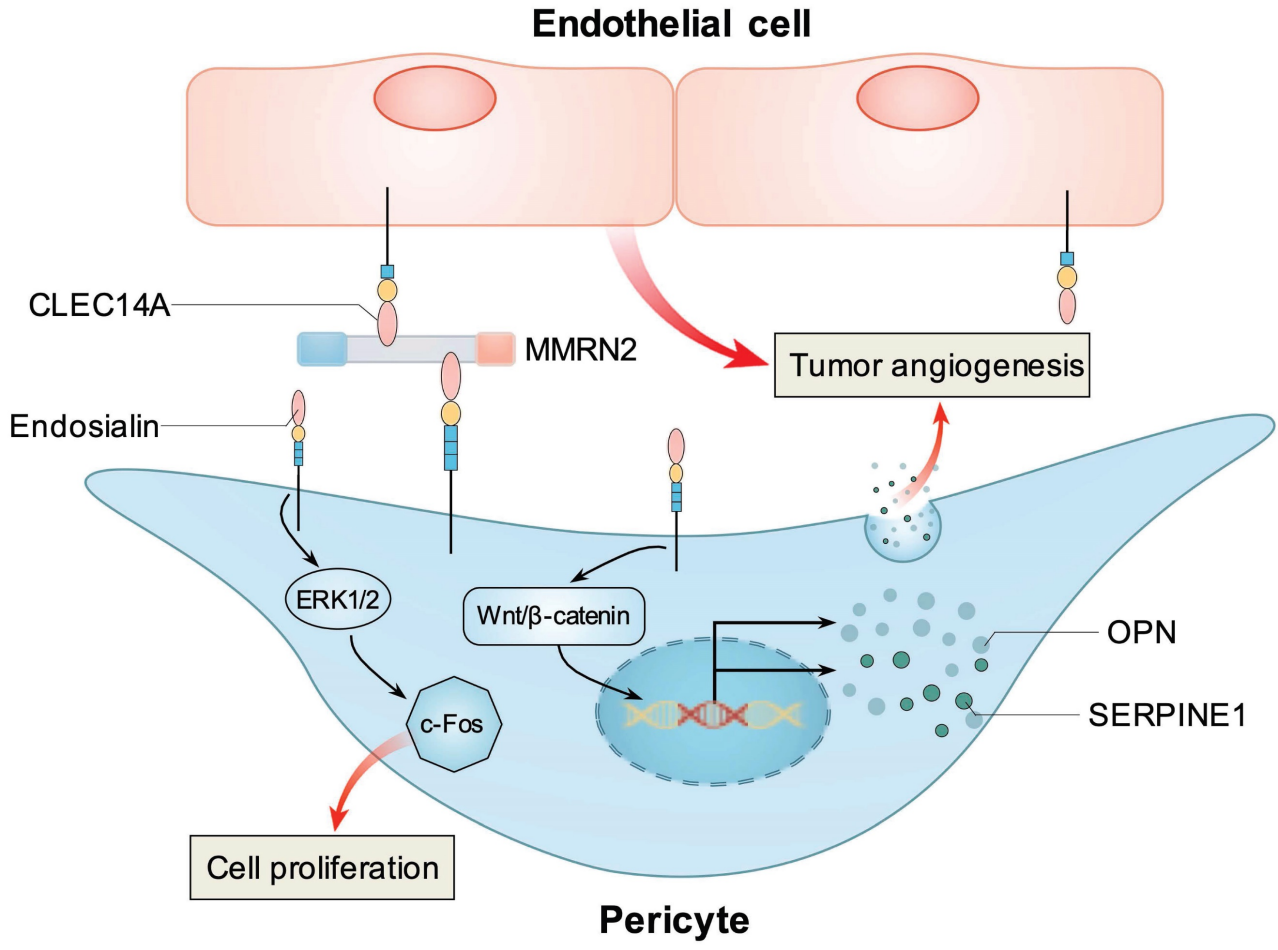
** Known mechanisms how endosialin stimulates tumor angiogenesis.** Pericyte expressed endosialin and endothelial cell expressed CLEC14A simultaneously bind with MMRN2 at the interface between the endothelium and pericytes to promote tumor angiogenesis. Endosialin could activate ERK1/2/c-Fos pathway to promote pericytes proliferation. Endosialin could activate Wnt/β-catenin signaling and upregulate two angiogenic factors, OPN and SERPINE1, in pericytes to promote tumor angiogenesis.

These findings indicate that endosialin is involved in tumor angiogenesis, and known mechanisms how endosialin stimulates tumor angiogenesis were summarized in Figure 2. Because of its role in tumor angiogenesis, endosialin is also considered to be an effective target for antiangiogenic therapy in different cancer types 62, 63.

### 3.3 Inducing an immunosuppressive tumor microenvironment

Cancer progression depends on the surrounding TME. Endosialin is expressed in CAFs and pericytes, which are important components of the TME and contribute to the formation of an immunosuppressive TME, thus promoting tumor progression.

In hepatocellular carcinoma (HCC), Yang et al. found that endosialin-positive CAFs could recruit macrophages through interaction with CD68 and promote M2 polarization by inducing CAFs to secrete Gas6, thus promoting HCC progression 64. In non-small cell lung cancer (NSCLC), Wu et al. reported that endosialin-positive CAFs could secrete CXCL12 to mediate the M2 polarization of macrophages both *in vitro* and *in vivo*, thus promoting NSCLC progression 65.

In RCC, Zhang et al. found that high endosialin expression was closely associated with patients' poor prognosis and immunosuppressive TME, such as increased infiltration of regulatory T cells (Treg) and upregulated immune checkpoint molecules such as PD-1, CTLA-4 and LAG-3 66. Further study confirmed that the number of endosialin-positive CAFs was closely correlated with patients' poor prognosis and immunosuppressive TME, as indicated by increased exhausted T cells and M2 macrophages 29. Lu et al. found that high endosialin expression was associated with low cytotoxic T lymphocyte (CTL) infiltration in RCC tissues in both clinical patients and endosialin KO mice, and antibody blockade of endosialin promoted CTL infiltration and inhibited RCC growth *in vivo*. By using co-culture assay, they showed that knockdown of endosialin in pericytes or antibody blockade could promote T cell migration, thus they concluded that endosialin positive pericytes could promote tumor progression through inhibiting CTL infiltration. In addition, they also found that combined treatment with endosialin antibody and PD-1 antibody could enhance the antitumor efficacy of PD-1 antibody in an RCC xenograft model 67.

In glioma, Ochs et al. found that FACS-sorted human malignant glioma-derived pericytes (HMGPs) specifically express endosialin. These pericytes accumulated in human gliomas, and the levels of HMGPs and CD8^+^ T cells were negatively correlated. In addition, HMGPs could suppress T-cell responses *in vitro*, indicating that they could promote local immunosuppression in glioma 68.

**Figure 3:**
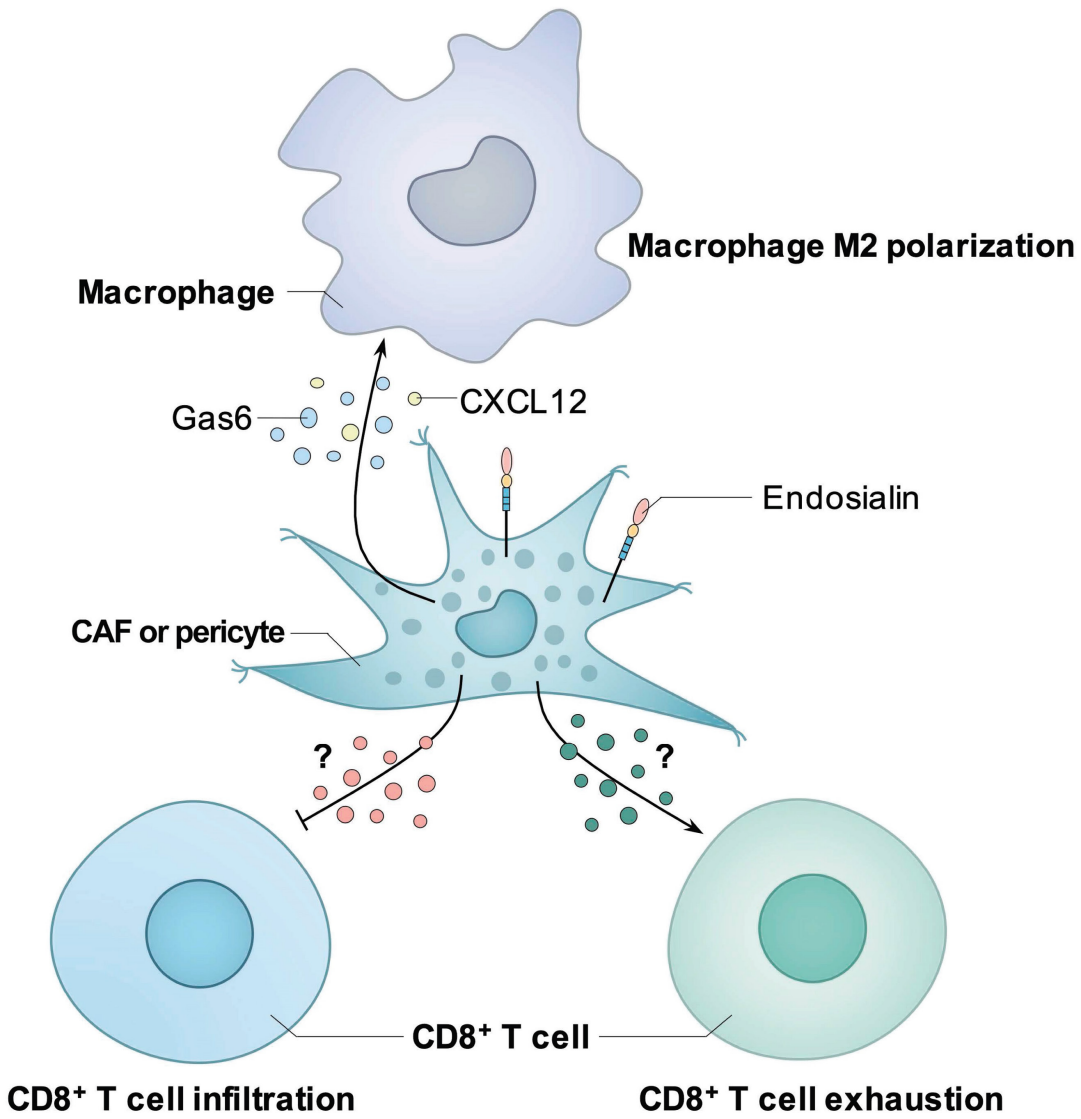
** Known mechanisms how endosialin induces immunosuppressive tumor microenvironment.** Endosialin-positive CAFs could secrete Gas6 or CXCL12 to mediate the M2 polarization of macrophages. Endosialin-positive CAFs or pericytes could inhibit the infiltration or induce the exhaustion of CD8^+^ T cell through unknown mechanisms.

Thus, endosialin can induce an immunosuppressive TME, probably through the regulation of the infiltration and exhaustion of CD8^+^ T cells or the recruitment and polarization of M2 macrophages (summarized in Figure 3). However, further studies are still needed to elucidate the detailed mechanisms.

## 4. Endosialin-targeted antitumor therapeutic strategies

Because of its tumor-promoting function, endosialin is considered an effective antitumor target. To date, a variety of endosialin-specific antibodies have been developed, and different endosialin-targeted strategies have been designed (Table 1).

### 4.1 Endosialin-specific antibodies

MORAb-004 (also called ontuxizumab), the humanized version of the mouse anti-human endosialin antibody Fb5, was the first antibody that was applied for cancer therapy in clinical trials. In 2014, the first phase I trial was conducted in 36 patients with treatment-refractory solid tumors. Patients were treated at 10 dose levels (ranging from 0.0625 to 16 mg/kg) once a week in 4-week cycles, and it was found that MORAb-004 was safe when the dose was up to 12 mg/kg, and preliminary antitumor activity was observed 69. Norris et al. conducted a phase I trial in children with relapsed or refractory solid tumors and found that MORAB-004 was well tolerated when administered weekly at 12 mg/kg 70. Doi et al. performed another phase I trial in Japanese patients with solid tumors who had failed standard chemotherapy, and they observed long-term disease stabilization in gastric cancer (GC) and extraskeletal chondrosarcoma, and tumor shrinkage in gastrointestinal stromal tumor (GIST) and HCC. The maximum tolerated dose (MTD) was not reached, and they recommended 8 mg/kg weekly or 12 mg/kg biweekly for further application 71.

In 2018, D'Angelo et al. performed a phase II trial in 76 patients with metastatic melanoma. Patients were given 2 or 4 mg/kg MORAb-004 weekly, and MORAb-004 was found to be well tolerated at both doses. The 24-week progression-free survival (PFS) value was 11.4% among all treated patients, and the overall response rate (ORR) was 3.1% at the 4 mg/kg dose, with clinical benefit achieved in 42.4% of response evaluable patients 72. Another phase II trial was performed in 126 chemorefractory metastatic colorectal cancer patients. Patients were given intravenous MORAb-004 (8 mg/kg) weekly or placebo plus best supportive care. Although MORAb-004 was found to be well tolerated, there were no significant differences between MORAb-004 monotherapy and placebo in terms of PFS, overall survival or ORR 73. In another phase I and randomized controlled phase II trial, Robin et al. examined the safety and efficacy of the combined treatment of MORAb-004 with gemcitabine and docetaxel (G/D) in 209 patients with metastatic soft-tissue sarcomas. They found that although the combination of MORAb-004 plus G/D was generally well tolerated, no significant difference was observed in either PFS or median overall survival between the MORAb-004 plus G/D group and the placebo plus G/D group 74.

It seemed that MORAb-004 alone was not sufficient to elicit an effective antitumor effect. The inefficiency of MORAb-004 might be caused by the fact that MORAb-004 does not have antibody-dependent cell-mediated cytotoxicity (ADCC) and complement-dependent cytotoxicity (CDC) effects, which are important for the antitumor effects of many antibodies 54. However, since it can internalize into endosialin-positive cells, it is possible to elicit antitumor effects if using this antibody to deliver cytotoxic drugs via different strategies, such as antibody‒drug conjugates (ADCs). For example, the CD30 antibody showed limited activity as a single agent, but after conjugation with monomethyl auristatin E (MMAE) (named Brentuximab Vedotin), the ADC showed potent antitumor effects and has been approved by the FDA for clinical application 75, 76. Another option for improving its antitumor effect is to combine it with other therapeutic treatments. For example, the combination of rituximab with CHOP chemotherapy showed an increased complete response rate and prolonged event-free and overall survival, without a significant increase in toxicity compared to the CHOP alone group 77. Since specific expression of endosialin in the tumor stroma of many cancers and also tumor cells in sarcomas have been well demonstrated, we believe that other endosialin-specific antibodies or antibody-based therapeutic strategies deserve further investigation.

Currently, in addition to MORAb-004, several other antibodies targeting endosialin have also been developed. For example, Zhao et al. obtained an endosialin-specific single chain antibody fragment (scFv78) by screening novel paired display-secretory yeast libraries. The binding affinity, specificity, and *in vivo* distribution of scFv78 were validated by flow cytometry, ELISA and immunofluorescence staining, which indicates that it could be used for endosialin-targeted imaging and therapy 78. Fierle et al. screened two new endosialin scFvs (1C1m and 7G22) from a phage display antibody library, and their group examined their application in different therapeutic strategies (discussed in the following section) 79.

O'Shannessy et al. immunized female Lewis rats and generated a series of novel monoclonal antibodies (mAbs) that can recognize different extracellular domains of endosialin. Through pairing different antibodies to setup an electrochemiluminescence (ECL) assay, they demonstrated that these antibodies could be used to detect soluble endosialin/TEM-1 (sEND) in the serum of CRC patients and healthy individuals 31. In addition, other endosialin-specific antibodies, such as ScFv-CM6 and hMP-E-8.3, were also generated and applied in different strategies (shown in the following section).

### 4.2 Other endosialin-targeted therapeutic strategies

MORAb-004 was the only endosialin antibody that was applied in clinical trials; however, it seemed that it could not elicit an effective antitumor effect when applied alone; thus, researchers have tried to pursue other endosialin-targeted therapeutic strategies, such as radioimmunotherapy, ADCs, CAR-T (chimeric antigen receptor T cell), BiTEs (bispecific T-cell engagers), immunotoxins, immunoliposomes, nanoparticles, and even DNA vaccines.

For radioimmunotherapy, D'Onofrio et al. evaluated a new panel of endosialin antibody fragments obtained from a phage display antibody library. They identified that the ^125^I radiolabeled antibody fragment 1C1m-Fc, which contains the endosialin-specific scFv (1C1m) and Fc, had high affinity for both human and mouse endosialin, could be effectively internalized into endosialin-positive cells, and could be specifically distributed in A673 xenografts in mice. Thus, it could be a promising candidate for the development of endosialin-targeted radioimmunoconjugates 80. Delage et al. conjugated 1C1m-Fc to p-SCN-Bn-DOTA and labeled it with ^177^Lu and confirmed that it could be specifically taken up by endosialin-positive tumors through biodistribution and single-photon emission SPECT/CT imaging studies 81. Later, they also examined the best DOTA per 1C1m-Fc ratio for theranostic applications and found that one DOTA per 1C1m-Fc gave the best pharmacokinetic behavior for future application of ^177^Lu-1C1m-Fc in patients 82.

For ADCs, Capone et al. generated a kind of ADC that contained the endosialin antibody hMP-E-8.3 (a humanized antibody) and a duocarmycin derivative. The ADC showed powerful, specific and target-dependent killing activity *in vitro* and led to long-lasting tumor growth inhibition in a cell line-based human osteosarcoma model. These results demonstrated that endosialin is an attractive target in sarcoma and that endosialin-specific ADC has the potential to be developed into a biotherapy agent for these malignancies 83. Rouleau et al. conjugated a human anti-endosialin antibody with the anti-neoplastic agent MMAE through the maleimidocaproyl-valine-citrulline-p-aminobenzylcarbamate linker (so-called endosialin-MC-VC-PABC-MMAE) and demonstrated that the ADC had selective cytotoxicity to endosialin-positive cells *in vitro* and achieved profound and durable antitumor efficacy in human tumor xenograft models 84.

For T-cell-mediated immunotherapy, Fierle et al. constructed two types of CAR-T cells and soluble bispecific trivalent engagers, which they termed TriloBiTEs (tBs), by using two endosialin scFvs (1C1m and 7G22). They demonstrated that the two types of CAR-T cells could specifically recognize endosialin-positive target cells and be activated; the tBs could redirect T cells toward endosialin-positive target cells, and systemic delivery of 1C1m-tB could effectively prevent the establishment of Ewing sarcoma tumors in a xenograft model. These data further confirmed that endosialin could be used as a promising target for T-cell-mediated immunotherapy 79.

For immunotoxin, Guo et al. generated an immunotoxin through conjugating the anti-endosialin 78Fc, which contains scFv78 and Fc fragment, with saporin (78Fc-Sap) and confirmed that 78Fc-Sap was effective in killing endosialin-positive sarcoma cells *in vitro* and could eliminate human sarcoma xenografts without apparent toxicity *in vivo* 32.

For immunoliposomes, Marty et al. isolated a single chain antibody fragment (scFv-CM6) that specifically binds to the extracellular part of endosialin using phage display technology. They further functionalized and coupled ScFv-CM6 to liposomes to generate immunoliposomes and loaded them with cytotoxic drugs, which showed increased binding affinity and up to 80% higher cytotoxic activity toward endosialin-expressing IMR-32 tumor cells compared with control liposomes 85.

For nanoparticles, Matthaiou et al. developed PEGylated poly (lactic-co-glycolic acid) (PLGA) nanoparticles (NPs) and functionalized them with the anti-endosialin antibody fragment 78Fc and loaded them with the necroptosis-inducing agent shikonin (SHK) (78Fc-PLGA-SHK NPs). They found that these NPs had significant toxicity *in vitro*, could effectively inhibit the growth of endosialin-positive tumors, and could induce an obvious immune response *in vivo* 86.

In addition to antibody-based therapy, endosialin was also targeted for cancer therapy through DNA vaccination. Facciponte et al. immunized immunocompetent mice with endosialin cDNA fused to the minimal domain of the C fragment of tetanus toxoid (referred to as the Tem1-TT vaccine) and demonstrated that Tem1-TT vaccination could elicit CD8^+^ and/or CD4^+^ T-cell responses, reduce tumor vascularity, increase CD3^+^ T-cell infiltration, and control the progression of established tumors. In addition, prophylactic immunization can prevent or delay tumor formation in several murine tumor models 87. Furthermore, combinatorial treatment may enhance the therapeutic effect of DNA-based cancer vaccines. For example, combination with chemotherapy may enhance immunogenicity, and combination with approaches to alleviate myeloid-derived suppressor cell (MDSC)- and Treg cell-induced immunosuppressive TME may also enhance the therapeutic effect of DNA vaccines 88.

### 4.3 Imaging and diagnostic strategies targeting endosialin

Because of its specific expression in tumor stroma in many cancers and also tumor cells in sarcomas, endosialin has also been used in tumor imaging or diagnosis. For example, Yuan et al. evaluated the potential application of scFv78 as a tool for tumor molecular imaging and found by optical imaging that scFv78 was specifically localized in tumors in a tumor mouse model that had highly endogenous mouse endosialin expression in the vasculature 89. Li et al. also confirmed that the radiolabeled fusion protein 78Fc could distinguish mouse- or human-endosialin-expressing tumor grafts from normal organs and control grafts *in vivo*. Thus, it could be further developed and optimized as an endosialin-targeted imaging agent for clinical application 90. Cicone et al. conjugated scFv78-Fc with the chelator p-SCN-Bn-CHX-A"-DTPA and then labeled the product with indium-111 to generate ^111^In-CHX-DTPA-scFv78-Fc. They demonstrated that it was stable in serum and could specifically bind with endosialin-positive cells and target endosialin-positive xenografts in tumor-bearing mice, providing translation potential for the diagnosis of sarcoma 91.

Chacko et al. radiolabeled MORAb-004 with ^124^I and confirmed its specific and sensitive binding with endosialin-positive cells and demonstrated that radiolabeled MORAb-004 could be specifically and sensitively taken up by endosialin-positive tumors *in vivo*, suggesting that it could be clinically used for immuno-PET to assess endosialin-positive tumor status 92. By using a tumor model that contained tumor cells and endosialin-expressing endothelial cells, Li et al. also demonstrated that ^124^I-labeled MORAb-004-PET could be used to visualize tumors enriched with endosialin-positive vasculature with high specificity and sensitivity 57.

These studies confirmed that endosialin is a promising target for the treatment and diagnosis of endosialin-positive sarcomas and multiple kinds of tumors with endosialin-positive stroma or vasculature.

## 5. Conclusion and perspectives

The identification of tumor-specific biomarkers could help to develop therapeutic strategies for targeted therapy, thus improving antitumor efficacy with minimal side effects. As a transmembrane glycoprotein, endosialin was found to be specifically and highly expressed in the stroma of various epithelial cell-derived tumors and both stroma and tumor cells of mesenchymal cell-derived sarcomas. Endosialin could promote tumor cell proliferation, adhesion and migration, stimulate tumor angiogenesis, and induce an immunosuppressive TME; thus, it is considered to be an ideal therapeutic target for cancer treatment.

Several anti-endosialin-specific antibodies have been developed, and various endosialin-targeted therapeutic strategies have been designed, some of which have shown preliminary antitumor effects. However, for the application of these endosialin antibodies or antibody-based therapeutic strategies in cancer treatment, several aspects need to be further validated. First, although the specific expression and tumor-promoting function of endosialin are relatively clear, the detailed mechanisms by which it promotes tumor progression and whether endosialin antibodies or antibody-based therapeutic strategies inhibit these mechanisms still need to be further elucidated. Second, some studies have found that endosialin could be detected in the serum of cancer patients, especially colon cancer patients, and whether patients with other cancer types also have serum endosialin expression and whether serum endosialin may influence the therapeutic effect of antibody-based therapies also need to be examined. In addition, the factors that regulate endosialin expression are not clear; although it has been found that the transcription factors HIF-2 and SP1 could regulate endosialin expression under hypoxia and at high cell density, the detailed mechanisms by which endosialin is regulated also need to be elucidated 93, 94. The clarification of its tumor-promoting mechanism and expression regulation mechanism will help to develop more efficient and specific endosialin-targeted therapeutic and diagnostic strategies and more effective combined therapeutic strategies for cancer treatment.

## Figures and Tables

**Figure 4:**
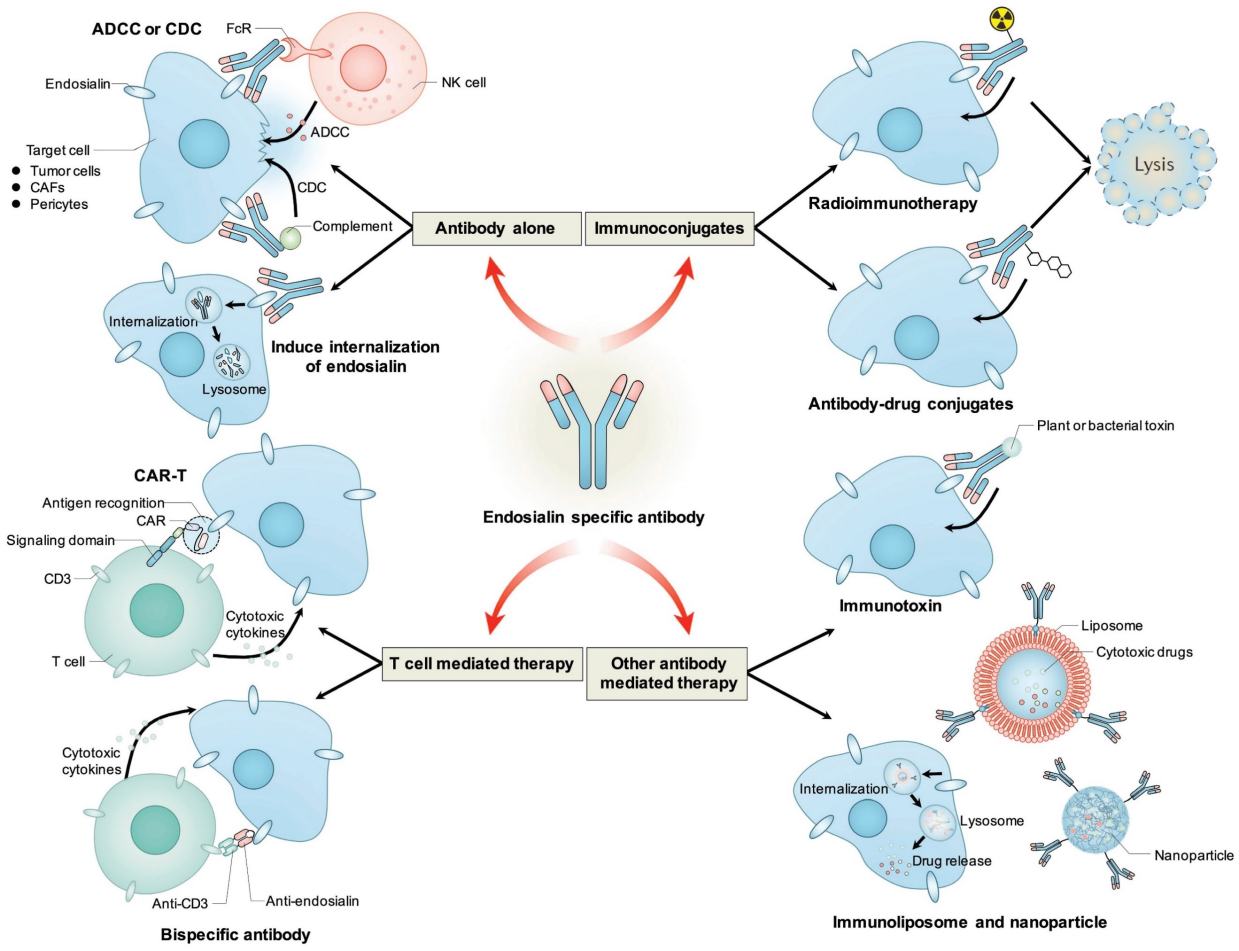
** Antibody based therapeutic strategies targeting endosialin.** Antibody alone could mediate ADCC or CDC effects to induce cytotoxicity to tumor cells, CAFs or pericytes, or induce the internalization of endosialin to inhibit its tumor-promoting function. Antibody could be conjugated with radioisotope or cytotoxic drugs and deliver them to endosialin positive cells to mediate specific cell killing. Antibody variable regions could be used to retarget immune effector cells towards endosialin positive cells through CAR-T or bispecific antibody. Antibody could also be fused with plant or bacterial toxin, or coupled to liposomes or nanoparticles to deliver toxin or cytotoxic drug to induce specific cell killing.

**Table 1:** Endosialin specific antibodies and antibody based endosialin-targeted anti-tumor therapeutic strategies.

Antibody name	Antibody type	Antibody source	Current stage	Therapeutic strategies	Patients or xenograft models	Refs
MORAb-004 (Ontuxizumab)	humanized antibody (IgG1)	Mouse immunized with human endosialin protein	Phase I	Antibody alone (dose ranging from 0.0625 to 16 mg/kg)	Treatment-refractory solid tumors	69
				Antibody alone (12 mg/kg)	Relapsed or refractory solid tumors (children)	70
				Antibody alone (8 mg/kg weekly or 12 mg/kg biweekly)	Solid tumors (Japanese patients)	71
			Phase II	Antibody alone (2 or 4 mg/kg, weekly)	Metastatic melanoma	72
				Antibody (8 mg/kg) plus best supportive care	Chemo-refractory metastatic colorectal cancer	73
				Antibody (8 mg/kg) combined with gemcitabine and docetaxel (G/D)	Metastatic soft-tissue sarcomas	74
1C1m-Fc	Fully human scFv + Fc			Radioimmunotherapy (radiolabeled with ^125^I or ^177^Lu)	Ewing's sarcoma A-673 or neuroblastoma SK-N-AS xenograft model	80-82
hMP-E-8.3	Humanized antibody	Mouse immunized with extracellular endosialin peptides	Preclinical	ADC (conjugated with duocarmycin derivative)	Osteosarcoma SJSA-1 xenograft model	83
Not named anti-endosialin	Fully human antibody	Humanized transgenic mouse	Preclinical	ADC (conjugated with MMAE)	Neuroblastoma SK-N-AS and Ewing's sarcoma A-673 xenograft model	84
1C1m, 7G22	Fully human scFv	Phage display library	Preclinical	CAR-T, TriBiTEs	Ewing's sarcoma A-673 xenograft model	79
scFv78-Fc	Fully human scFv + Fc	Yeast display library	Preclinical	Immunotoxin (78Fc and saporin)	Osteosarcoma SJSA-1 and Ewing's sarcoma A-673 xenografts model	32
				PLGA nanoparticles (loaded with shikonin)	Endosialin-expressing MS1 and TC1 xenograft model	86
ScFv-CM6	Fully human scFv	Phage display library	Preclinical	Immunoliposome (loaded with cytotoxic drug)	Endosialin-expressing IMR-32 xenograft model	85

## Abbreviations

ADC: antibody-drug conjugates
α-SMA: α-smooth muscle actin
BiTEs: bispecific T-cell engagers
CAFs: cancer associated fibroblasts
CAR-T: chemeric antigen receptor T-cell
Col I: collagen type I
CRC: colorectal cancer
CTL: cytotoxic T lymphocyte
DOTA: dodecane tetraacetic acid
ECM: extracellular matrix
EPC: endothelial precursor cells
FN: fibronectin
GC: gastric cancer
HCC: hepatocellular carcinoma
MDSCs: myeloid-derived suppressor cells
NPs: nanoparticles
NSCLC: non-small cell lung cancer
ORR: overall response rate
OS: osteosarcoma
PDGFR-β: platelet-derived growth factor receptor-β
PEG: polyethylene glycol
PET: positron emission tomography
PFS: progression-free survival
PLGA: poly (lactic-co-glycolic acid)
RCC: renal cell carcinoma
SP: side population
TEM1: tumor endothelial marker 1
TME: tumor microenvironment
Treg: regulatory T cells
UCB: urothelial carcinoma of the bladder
